# COGNITIVE TRAINING APPROACHES TO IMPROVE COGNITION AND ASSOCIATED FUNCTIONS IN MID TO LATE LIFE

**DOI:** 10.1093/geroni/igad104.1203

**Published:** 2023-12-21

**Authors:** Ashley Curtis, Amy Costa, Madison Musich, Chloe Rzeppa, Christina McCrae

**Affiliations:** University of South Florida, Tampa, Florida, United States; University of South Florida, Tampa, Florida, United States; University of Missouri-Columbia, Columbia, Missouri, United States; University of Missouri, Columbia, Missouri, United States; University of South Florida, Tampa, Florida, United States

## Abstract

Cognitive Training (CT) has yielded inconsistent benefits in non-patient populations. Despite known cognition/sleep associations, limited studies have investigated CT’s impact on sleep. We examined cognitive and associated outcomes across three CT programs in older adult (OA) insomnia patients [with/without mild cognitive impairment (MCI)] and middle-aged adults (MA) with generalized anxiety disorder (GAD). In Study 1, OAs with insomnia (Mage=67.9±8.0; 16 women/8 men) completed 6-week CT (Nintendo DS-Big Brain Academy; 60 mins/day, 3x/week) and waitlist control (WLC) in a cross-over design. At baseline and post-CT/post-WLC, participants completed NIH toolbox-cognitive tasks. In Study 2, MAs with GAD (Mage=49.8±4.0; 29 women) underwent 8-week CT (Cognifit, n=14) or Trivia Training (TT, n=15), 45 mins/day, 3x/week and completed pre/post Inquisit-cognitive assessments. In Study 3, OAs with insomnia and MCI (Mage=67.6±8.0; 10 women/3 men) completed 8-week CT (Cognifit; 45 mins/day, 3x/week) and pre/post Cambridge Brain Sciences-cognitive and polysomnography-sleep assessments. In Study 1, proportional RAVLT-verbal memory improvement was larger [t(23)=2.01, p=.028] following CT(17%) versus WLC(9%). Similarly, proportional Flanker-inhibition improvement was larger [t(23)=1.71, p=.05] following CT(2%) versus WLC(-.3%). In Study 2, CT improved Posner-attention orienting more than TT [F(1,27)=5.25, p=.03]. In Study 3, CT improved Digit-span-working memory [t(12)=-1.84, p=.045], reduced number of awakenings [t(12)=2.70, p=.01), increased %REM [(t(12)=-1.77,p=.05], and showed trending improvement for spatial planning/reasoning [t(12)=1.70, p=.057] and %Lighter-sleep [t(12)=1.62,p=.066]. CT benefits non-trained cognition (transfer effects) in mid-to-late life individuals with insomnia, GAD and MCI, and shows promise for improving sleep in MCI. Follow-up in larger samples investigating mechanisms underlying CT effects (domain-specific training, sleep improvement) is warranted.

